# The single-use rhinolaryngoscope: an evaluation and cost comparison

**DOI:** 10.1017/S0022215120001656

**Published:** 2020-09

**Authors:** R Mistry, R V Russell, N Walker, E Ofo

**Affiliations:** 1 Department of Otolaryngology, St George's University Hospitals NHS Foundation Trust, London, UK; 2 Ambu A/S, Ballerup, Denmark

**Keywords:** Endoscopy, Otolaryngology, Costs And Cost Analysis

## Abstract

**Background:**

This study investigated whether the single-use rhinolaryngoscope is clinically and economically comparable to the conventional reusable rhinolaryngoscope within a tertiary otolaryngology centre in the UK.

**Methods:**

A non-blinded, prospective and single-arm evaluation was carried out over a 5-day period, in which micro-costing was used to compare single-use rhinolaryngoscopes with reusable rhinolaryngoscopes.

**Results:**

Overall, 68 per cent of the investigators perceived the single-use rhinolaryngoscope to be ‘good’ or ‘very good’, while 85 per cent believed the single-use rhinolaryngoscope could replace the reusable rhinolaryngoscope (*n* = 59). The incremental costs of reusable rhinolaryngoscope eyepieces and videoscopes in the out-patient clinic, when compared to single-use rhinolaryngoscopes, were £30 and £11, respectively. The incremental costs of reusable rhinolaryngoscope eyepieces and videoscopes in the acute surgical assessment unit, when compared to single-use rhinolaryngoscopes, were −£4 and −£73, respectively.

**Conclusion:**

The single-use rhinolaryngoscope provides a clinically comparable, and potentially cost-minimising, alternative to the reusable rhinolaryngoscope for use in the acute surgical assessment unit of our hospital.

## Introduction

Endoscopy allows for the enhanced visualisation, inspection, manipulation and treatment of internal organs or tissues, without the need for an incision.^1^ The endoscope has revolutionised otolaryngology by virtue of its ability to directly visualise the nose, throat and airway, in an emergency, in-patient or out-patient setting. There are a range of indications for the use of an endoscope in otolaryngology, both in emergencies and non-emergencies, including airway obstruction, foreign body removal, hoarseness, globus sensation, recurrent epistaxis, cancer surveillance, evaluation of obstructive sleep apnoea, fibre-endoscopic evaluation of swallowing, and assessment and treatment of vocal fold lesions.^2^

In current practice, once an endoscope has been utilised on a single patient, it is required to undergo reprocessing, as required by the Health and Social Care Act.^3,4^ If contaminated, flexible endoscopes pose a moderate degree of infection risk, and therefore require ‘high-level disinfection’ to eliminate vegetative bacteria, mycobacteria, fungi and viruses. The importance of this practice is underpinned by the emergence of multi-drug resistance, and organisms such as mycobacteria, bacterial spores, Creutzfeldt–Jakob disease (CJD) and variant CJD.^5^ Accordingly, the 2015 risk assumptions put forward by the UK Advisory Committee for Dangerous Pathogens and Health Technical Memorandum 01-01 recommend that if a patient with an undiagnosed neurological illness undergoes an invasive endoscopy, where variant CJD cannot be excluded, or where the sub-classification of CJD infection is still pending, then it is necessary to place the device into temporary quarantine.^5,6^ Unless the potential variant CJD contamination can be subsequently rescinded, then the quarantined endoscope cannot be returned to normal use on other patients.^5,6^

ENT UK provides national guidelines for endoscope decontamination.^7^ Decontamination can take the form of chemical decontamination (e.g. wipe systems such as chlorine dioxide) or central decontamination systems. Chemical decontamination is less expensive but is deemed an inferior method of decontamination.^7^ Central decontamination has a higher cost, and ENT UK guidelines state that hospitals considering central decontamination models should be aware of the significant cost implications of such models. While the risk of cross-infection and harm remains low, the consequence of prion transmission remains a serious potential risk. ENT UK concurs with the advice previously described by the UK Advisory Committee for Dangerous Pathogens and Health Technical Memorandum 01-06 regarding variant CJD risk and endoscope decontamination.^7^ Every hospital or clinic should maintain a robust system of individual endoscope traceability, with regular audit. In particular, it states that hospitals should consider disposable flexible endoscopes available for use in patients with variant CJD.^7^

At the time of submission, the coronavirus disease 2019 (Covid-19) pandemic has provided further emphasis on the importance of thorough infection control during endoscopic examination, which is deemed to be an aerosol generating procedure. ENT practitioners themselves are deemed to be at high risk of Covid-19 transmission. ENT UK have provided clear guidance that endoscopy be carried out by video monitoring on all patients (regardless of Covid-19 status) where possible, rather than direct viewing through an eyepiece.^8^ Clear guidance is also provided for the decontamination of reusable scopes if used, which involves full donning of personal protective equipment and a specific decontamination procedure.^8^

Inadequate documentation of patients’ details and their relative risk of infection can therefore lead to the disposal of unnecessary reusable endoscopes and a consequential increase in capital expenses.^9,10^ Even if adequately documented, reprocessing can cost between £41 and £124 per procedure; moreover, the homogeneity and quality of the reprocessing practice to ensure ‘high-level disinfection’ cannot always be guaranteed.^11^

Rhinolaryngoscopes are currently available as rigid scopes, eyepiece scopes and videoscopes. Although rigid rhinolaryngoscopes are primarily used in operating theatres, eyepiece scopes and videoscopes are commonly used for procedures in both out-patient and in-patient consultations. The eyepiece scope allows visualisation through an eyepiece at the end of the device, allowing only the user to visualise the resultant image. In contrast, the videoscope allows the picture to be visualised on a monitor stack. Once used, both of these items of equipment should undergo the relevant decontamination process prior to subsequent use, and therefore are deemed reusable rhinolaryngoscopes.^3^

**Fig. 1.**
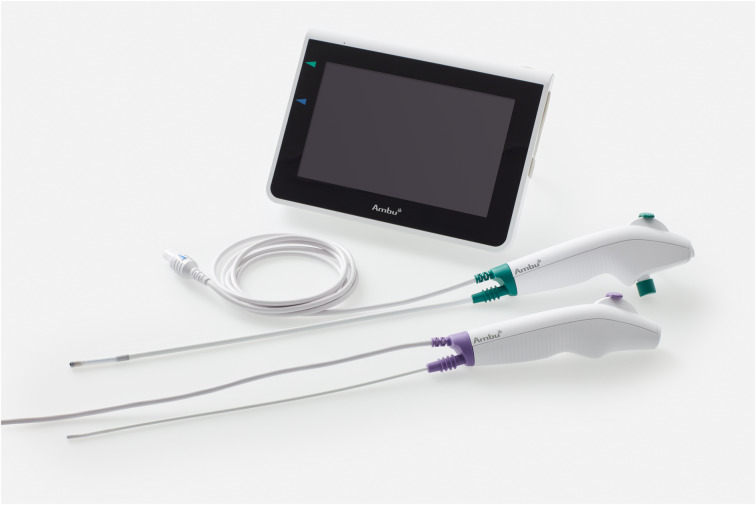
Image of the Ambu aScope 4 RhinoLaryngo Slim and Intervention scopes along with the Ambu aView (monitor).

The Ambu® aScope^™^ 4 RhinoLaryngo (Figure 1) is a single-use video endoscope that is available with and without a working channel. The Ambu aScope 4 RhinoLaryngo Slim (single-use rhinolaryngoscope), without a working channel, is intended for routine examinations of the upper airway anatomy (Figure 1).^12^ Immediately after patient use, the single-use rhinolaryngoscope is disposed of into a clinical waste bin. This practice prevents any transmission of infectious agents between patients, and reduces the costs associated with reprocessing.

Previous research with a single-use bronchoscope, the Ambu aScope 4 Broncho, clearly demonstrated the positive organisational impact created by a single-use device, reducing the number of support processes needed to ensure the provision of a clean and ready-to-use bronchoscope.^13^ Furthermore, the single-use bronchoscope has been proven to reduce the delay between the indication for bronchoscopy and the initiation of the actual procedure, while having a comparable environmental impact compared to reusable bronchoscopes.^14,15^ Although there is limited published evidence to support the use of single-use rhinolaryngoscopes, it is likely that the benefits of single-use bronchoscopes will also apply to single-use rhinolaryngoscopes as the reprocessing procedure is similar.^9,16^ However, the current lack of evidence prevents evidence-based decisions on whether the implementation of single-use rhinolaryngoscopes is clinically and economically practical.

We aimed to investigate whether the single-use rhinolaryngoscope is clinically comparable to the conventional reusable rhinolaryngoscope, and to compare the cost of introducing single-use technology as an alternative to the conventional reusable rhinolaryngoscope across different clinical settings.

## Materials and methods

A single-arm, non-blinded, prospective trial and cost-comparison exercise was carried out at a tertiary otolaryngology centre in the UK. The trial was conducted from 24th to 28th June 2019. A pre-trial training session was held, lasting 30–60 minutes, to describe the intentions of the study, and to introduce investigators without previous experience to the single-use rhinolaryngoscope technology and monitor (the Ambu aView^™^).

At the training session, the investigators conducted procedures on manikins under the supervision of an Ambu clinical trainer. Throughout the trial duration, an Ambu representative was available onsite for technical support. The investigators were then asked to employ the single-use rhinolaryngoscope for clinical procedures within the device's intended purpose, and then complete a pre-printed evaluation form. A Likert scale was used to quantify a range of parameters and provide an evaluation of the equipment, including image quality, advancing, navigation, overall perception and ergonomics. The investigators were also asked whether they had to change from the single-use rhinolaryngoscope to a reusable rhinolaryngoscope during their clinical procedures.

The overall aim of the evaluation was to ascertain whether the single-use rhinolaryngoscope could replace the reusable rhinolaryngoscope, and whether the investigators had to change from the single-use system to the reusable system during each clinical procedure. The investigators answered the questions using either ‘yes’ or ‘no’ responses. The Likert scores were then transformed into a numerical score from 1 to 5. The results are shown as mean ± standard deviation values. Ordinary least squares regression analysis was subsequently conducted to determine if the evaluation scores influenced the investigators’ opinion as to whether the single-use rhinolaryngoscope could replace the reusable rhinolaryngoscope. The evaluation form for the Ambu aScope 4 RhinoLaryngo Slim single-use rhinolaryngoscope is shown in Figure 1 in the supplementary material, available on *The Journal of Laryngology & Otology* website.

Two investigators independently reviewed the evaluation forms for validity. As a result of this review, questionnaire numbers 13 and 61 were excluded from the final analysis. Questionnaire 13 was excluded because the scores provided did not match the investigator's written comments. Questionnaire 61 was excluded because the investigator used a different scope.

The cost-comparison analysis was based on a hospital setting with a short time perspective. The main endpoint was the incremental cost of the procedure when the single-use rhinolaryngoscope was used, in comparison with the conventional reusable eyepiece and video rhinolaryngoscope. The rigour of the base-case result was assessed via two-way sensitivity analysis, and a break-even point was calculated dependent on the volume of procedures.

Micro-costing was conducted on reusable rhinolaryngoscopes between 24th and 27th September 2019. Rhinolaryngoscopes were followed and tracked from the finalised procedure, through reprocessing, and until the initiation of a new procedure. All person-hours, single-use equipment, utilities and capital equipment utilised during the supporting processes were noted, and each was allocated a specific cost. Personnel costs were based on the mean wage for pay band 2.^17^ Capital costs were projected via the consumer price index to 2018 prices, and discounted at a rate of 3.5 per cent to account for time preference.^18,19^ The amortisation period was five years for capital equipment in the out-patient clinic, three years in the acute surgical assessment unit, and eight years for reprocessing capital equipment.^18^ Overhead costs were added at a rate of 20 per cent to account for additional capital, repair and reprocessing costs.^20^

The final dataset was then subdivided into five categories: (1) objective evaluation of equipment; (2) a comparative evaluation against the scope normally used on a day-to-day basis; (3) overall evaluation; (4) qualitative comments; and (5) cost analysis.

## Results

The single-use rhinolaryngoscope was used on 200 occasions by a total of 16 investigators (9 otolaryngology consultants, 6 otolaryngology registrars and 1 core surgical trainee).

The questionnaires were completed on 61 occasions, thus yielding a compliance rate of 30.5 per cent; this represented a mean of 3.7 ± 0.9 completed questionnaires per investigator. Single-use rhinolaryngoscopes were used in four types of documented procedures: nasendoscopy (53 procedures), laryngoscopy (1 procedure), pharyngolaryngoscopy (1 procedure) and nasal examination (1 procedure); in three cases, the exact procedure was not documented.

The evaluation questions and mean scores are displayed in Table 1. A mean of 3 per cent of participants changed from the single-use rhinolaryngoscope to a reusable rhinolaryngoscope. A mean of 85 per cent believed the single-use rhinolaryngoscope could replace the reusable rhinolaryngoscope. The single-use rhinolaryngoscope was rated above 2.50 in all domains (where 1 = ‘very poor’ or ‘much worse’, and 5 = ‘very good’ or ‘much better’).

**Table 1.** Evaluation questions and mean scores

Question	Likert scores* (mean ± SD)
How would you rate image quality during the procedure just performed?	3.92 ± 0.98
How would you rate navigation of aScope4 RhinoLaryngo Slim through the relevant area?	3.98 ± 0.91
How would you rate overall perception of quality & functionality of aScope 4 RhinoLaryngo Slim for the procedure performed?	3.86 ± 0.85
How would you rate advancing & navigation compared to the scope you normally use?	2.81 ± 0.95
How would you rate ergonomics in the handle compared to the scope you normally use?	2.88 ± 0.90
How would you rate image quality compared to the scope you normally use?	3.12 ± 1.01
During the procedure, did you have to change from aScope 4 RhinoLaryngo Slim to your normal scope?	Yes: 3 ± 18%
Do you consider that aScope 4 RhinoLaryngo Slim can replace your existing reusable scope for the procedure performed?	Yes: 85 ± 36%

Likert scores were based on 1 (‘very poor’ or ‘much worse’) to 5 (‘very good’ or ‘much better’). *Unless indicated otherwise. SD = standard deviation

### Objective evaluation

**Fig. 2.**
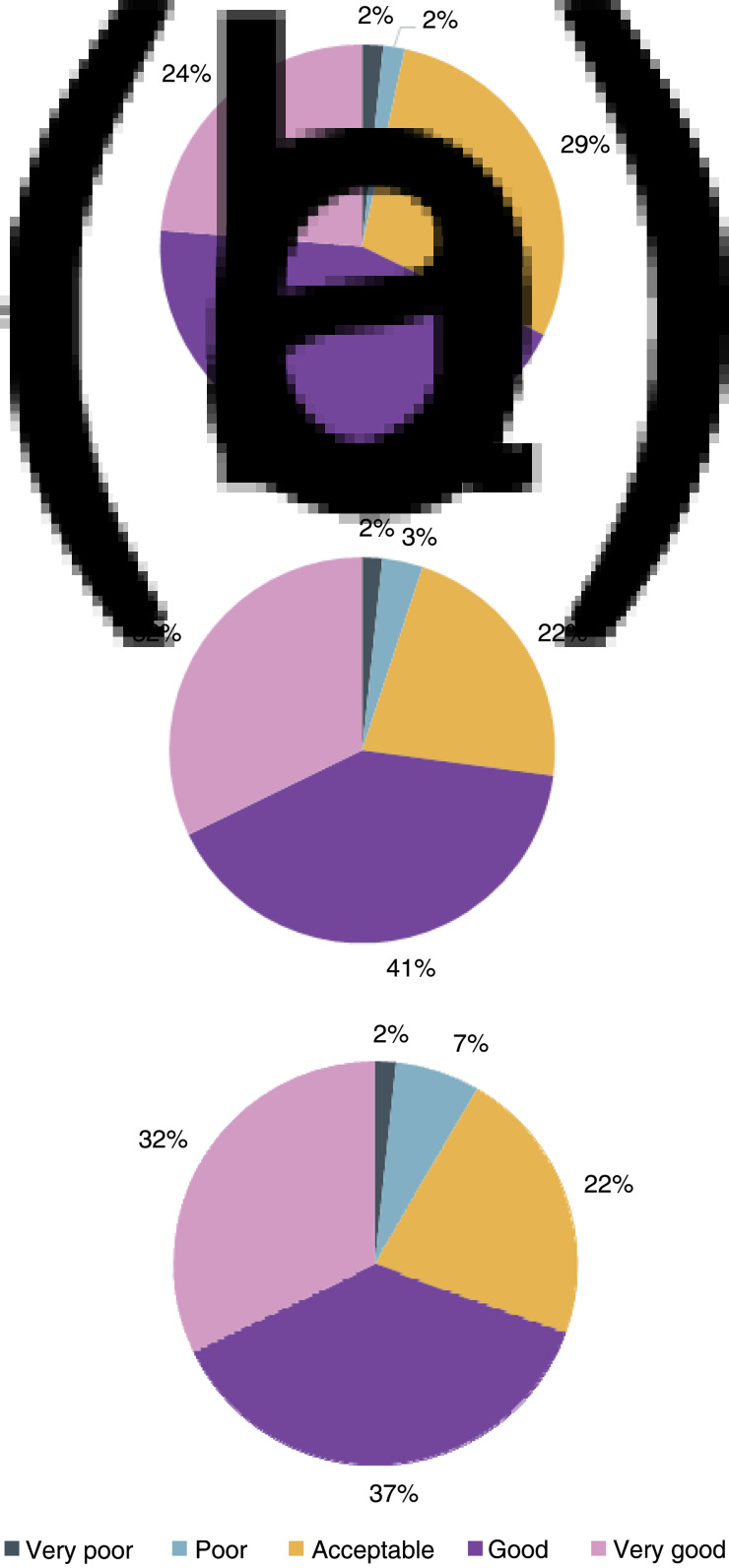
Objective evaluations of the (a) overall perception, (b) navigation and (c) image quality, of the single-use rhinolaryngoscope.

The results for overall perception, navigational ability and image quality can be seen in Figure 2. The single-use rhinolaryngoscope was deemed acceptable or better for overall perception, navigation and image quality by 96 per cent, 95 per cent and 91 per cent of participants, respectively.

### Single-use versus reusable rhinolaryngoscopes

**Fig. 3.**
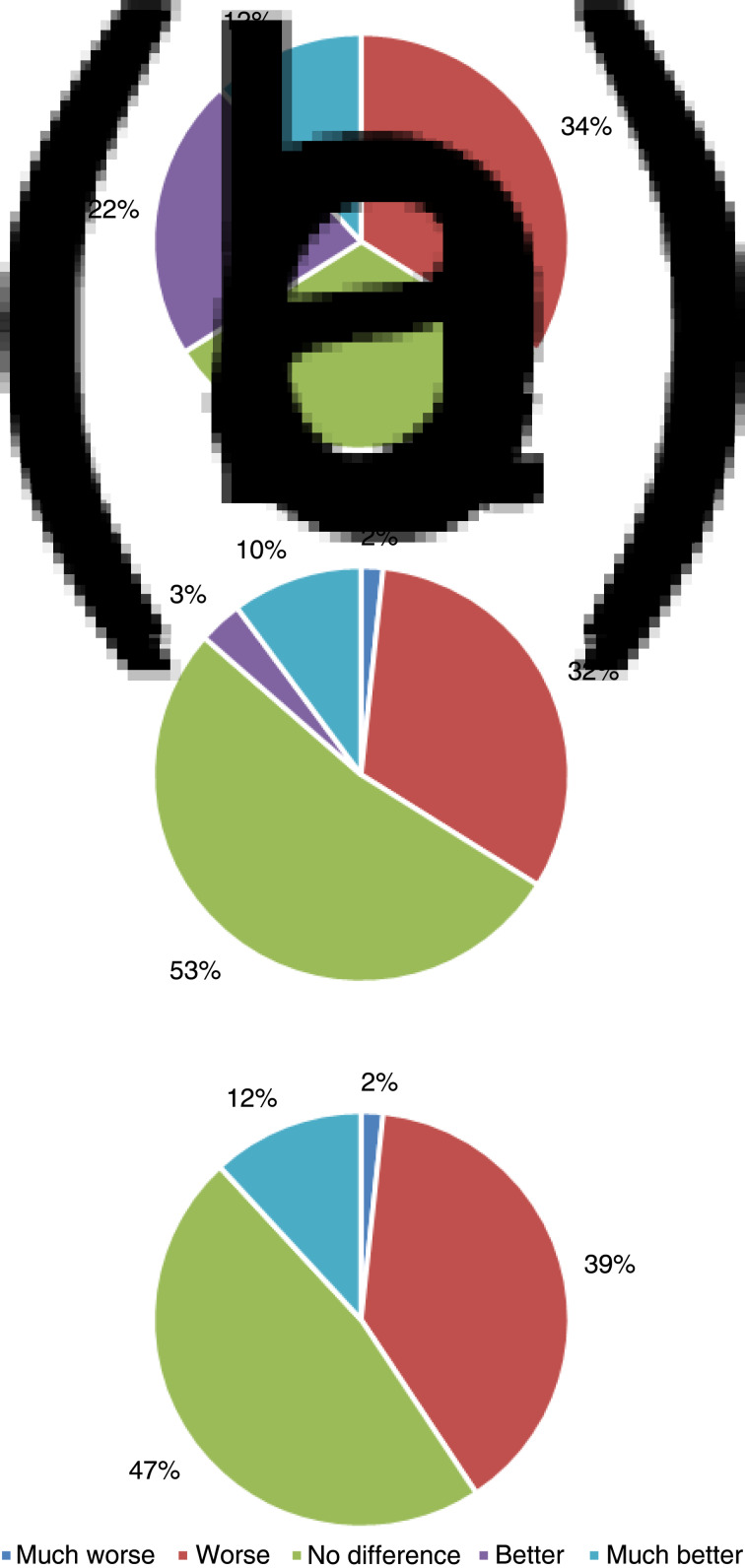
Evaluations of (a) image quality, (b) ergonomics, and (c) advancing and navigation of single-use rhinolaryngoscopes in comparison with reusable rhinolaryngoscopes.

The results comparing image quality, ergonomics, and advancing and navigation are shown in Figure 3. The single-use rhinolaryngoscope was deemed ‘better’ or ‘much better’ than the reusable rhinolaryngoscope for image quality, ergonomics, and advancing and navigation in 24 per cent, 13 per cent and 12 per cent, respectively. Participants found no difference between the single-use rhinolaryngoscope and the reusable rhinolaryngoscope for image quality, ergonomics, and advancing and navigation in 32 per cent, 53 per cent and 47 per cent, respectively.

### Overall evaluation

Overall, 3 per cent of the investigators reported that they had to change to the reusable rhinolaryngoscope because of patient intolerance. Eighty-five per cent of investigators believed that the single-use rhinolaryngoscope could successfully replace the reusable rhinolaryngoscope.

### Overall evaluation dependent on evaluation score

Overall, there was a significant association between the evaluation scores and an investigators' opinion as to whether the single-use rhinolaryngoscope could replace the reusable rhinolaryngoscope (F(6,52) = 5.02, *p* < 0.001). However, the only individual evaluation variable that had a significant effect on the investigator's decision that the single-use rhinolaryngoscope could replace the reusable rhinolaryngoscope was the evaluation of navigation (*t*(52) = 2.95, *p* = 0.005).

### Qualitative feedback

**Fig. 4.**
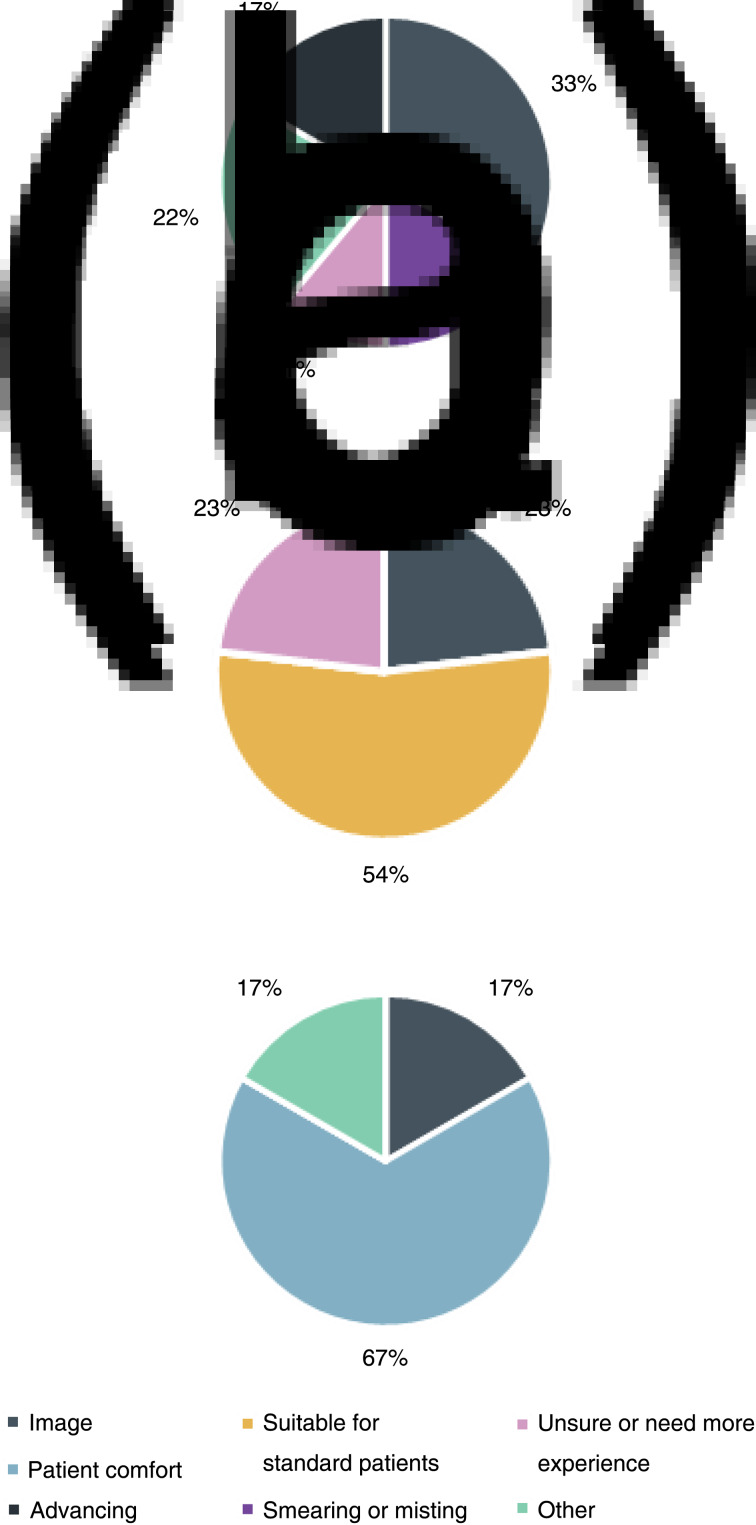
Categorisation of (a) negative, (b) neutral and (c) positive comments relating to the single-use rhinolaryngoscope.

In total, there were 25 qualitative feedback comments. This feedback was independently classified into three categories by two of the authors as negative, neutral or positive. The majority of the positive comments related to patient comfort, while most of the negative comments related to image quality. The neutral comments mostly related to the use of the scope in a ‘standard’ patient (deemed not to have complex anatomy or needs) (Figure 4).

### Cost analysis

In both the out-patient clinic and acute surgical assessment unit settings, the cost of a procedure performed using the single-use rhinolaryngoscope was £105. The base-case results were based on 4957 and 783 procedures per annum in the out-patient clinic and acute surgical assessment unit, respectively. The volume of procedures in the acute surgical assessment unit was based on an assumption that one-third of all otolaryngology patients undergo nasoendoscopy. The capital and repair cost input for the out-patient clinic cost analysis can be found in supplementary material Tables 1 and 2, available on *The Journal of Laryngology & Otology* website. The capital and repair cost input for the acute surgical assessment unit can be found in supplementary material Tables 3 and 4. Capital, consumable and utilities, and personnel times, for reprocessing reusable rhinolaryngoscopes can be seen in supplementary material Tables 5–7, respectively.

**Fig. 5.**
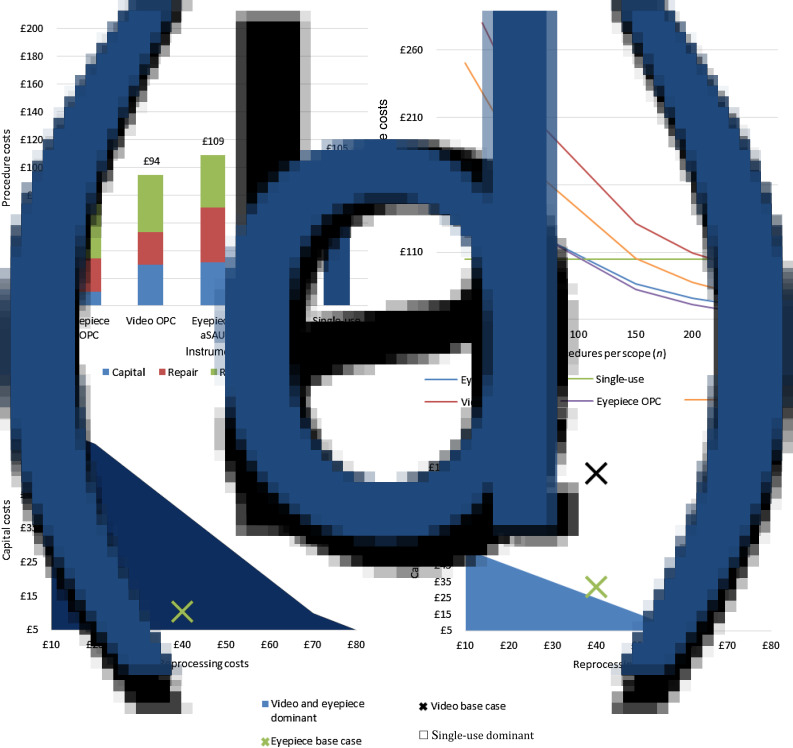
(a) The base-case for eyepiece, video and single-use rhinolaryngoscopes, in the out-patient clinic (OPC) and acute surgical assessment unit (aSAU). The total cost was segmented into capital, repair and reprocessing costs. (b) One-way sensitivity analysis for the cost of procedures, dependent on the volume of procedures each year. (c & d) Two-way sensitivity analysis showed dominant dependence on reprocessing and capital costs in the (c) out-patient clinic and (d) acute surgical assessment unit.

In the out-patient clinic, the cost of the eyepiece rhinolaryngoscope procedure was £75, including £10, £24 and £41, for capital, repair and reprocessing, respectively. In contrast, the cost of the video rhinolaryngoscope procedure in the out-patient clinic was £94, including £30, £23 and £41, for capital, repair and reprocessing, respectively. The incremental costs of reusable rhinolaryngoscope eyepieces and videoscopes in the out-patient clinic, when compared to single-use rhinolaryngoscopes, were £30 and £11, respectively. The break-even point occurred at 102 and 151 procedures per eyepiece and video rhinolaryngoscope, respectively (Figure 5).

In the acute surgical assessment unit, the cost of the eyepiece scope procedure was £109, including £32, £39 and £38, for capital, repair and reprocessing, respectively. The cost of the videoscope procedure in the acute surgical assessment unit was £178, including £101, £39 and £38, for capital, repair and reprocessing, respectively. The incremental costs of reusable rhinolaryngoscope eyepieces and videoscopes in the acute surgical assessment unit, when compared to single-use rhinolaryngoscopes, were −£4 and −£73, respectively. The break-even point occurred at 104 and 215 procedures for the eyepiece and video rhinolaryngoscope, respectively. Two-way sensitivity analysis was carried out for both settings, and showed that the base-case was sensitive to procedure volume, reprocessing and capital costs (Figure 5).

In order to ensure the provision of a ready-to-use reusable rhinolaryngoscope, 21 minutes of active personnel time was required. Furthermore, in the out-patient clinic, one dedicated member of staff was required, full-time, to ensure that reusable scopes were always available.

In the acute surgical assessment unit, all of the purchased reusable rhinolaryngoscopes were quarantined over a three-year period because of unrecorded procedures, or patient infection status; consequently, prion contamination could not be excluded (please see Tables 1–7 in the supplementary material, available on *The Journal of Laryngology & Otology* website, for all cost analysis data).

## Discussion

Overall, our analyses showed that the user experience and acceptability of the single-use rhinolaryngoscope was positive. This is in accordance with a previous evaluation carried out in Germany.^16^ Furthermore, our qualitative data demonstrated that the single-use rhinolaryngoscope was considered to be ‘acceptable’ or ‘better’ in more than 50 per cent of responses with regard to all three parameters (image quality, ergonomics, and advancing and navigation). Moreover, 85 per cent of participants believed that the single-use rhinolaryngoscope could replace the reusable system. In particular, qualitative comments from investigators revealed that patient comfort was deemed to be a particular advantage; typical comments included ‘patient found it more comfortable’ and ‘better tolerated, excellent images’.

### User acceptability: image quality

Non-comparative data relating to image quality were generally positive (69 per cent ‘good’ or ‘very good’); however, 33 per cent of the investigators rated the single-use rhinolaryngoscope as ‘worse’ or ‘much worse’ than the reusable system. This was in line with some of the qualitative feedback, which stated that ‘the optics are not as accurate/sharp as the traditional scope but still passable for the purpose required’. Another investigator stated ‘good enough for clinical out-patients or acute airway. Limited use for laryngeal detail’. These comments highlight the importance of the context in which the device is used.

Concerns were also raised regarding the monitor. For example, one investigator stated that ‘the optics were balanced by closing all the curtains, thus reducing reflection on the monitor’. Consequently, there is a need for the system to be used in an environment that allows good visualisation of the images shown on the monitor. Furthermore, this comment indicates an insufficient awareness of the possibility to adjust contrast settings on the aView monitor. Qualitative comments, such as ‘ability to use screen for recording/teaching/explanation is very useful’ indicate the additional advantages of having a monitor compared to the eyepiece; the quantitative data do not reflect this advantage.

### User acceptability: advancing and navigation

In total, 95 per cent of investigators scored navigation with the single-use rhinolaryngoscope as ‘acceptable’ or ‘better’ than that with the reusable rhinolaryngoscope. This was the only evaluated variable that had a significant effect on investigators’ perceptions of whether the single-use rhinolaryngoscope can replace the reusable rhinolaryngoscope. Comparative data further demonstrated that 59 per cent of investigators thought the single-use rhinolaryngoscope was ‘acceptable’ or ‘better’ than the reusable scope. Despite the preference towards the single-use rhinolaryngoscope, the qualitative data included negative comments, including ‘felt very uncomfortable as upside-down to normal position’, and ‘less flexible: navigation in a difficult nose may be difficult’.

All investigators, except for one core surgical trainee, had gained significant experience using the reusable rhinolaryngoscope throughout their careers. Navigation is likely to be associated with reversal learning and a renewal effect.^21^ Therefore, a more valid and comparative dataset for evaluating advancing and navigation could be acquired if reusable rhinolaryngoscopes and single-use rhinolaryngoscopes were provided to novices with similar levels of experience and over a longer period of time.

### User acceptability: patient selection

A common theme highlighted in the qualitative data was the importance of patient selection. Comments included ‘not replaceable for certain cases – should have the reusable scope available in case’, ‘had to swap to paediatrics scope as patient gagged’, and ‘difficult patients need reusable scope’. Hence, if a patient was deemed to have complex anatomy, or had a smaller airway, then the single-use rhinolaryngoscope would be less preferential than the reusable rhinolaryngoscope in light of the image quality and perceived navigation problems. However, our study did not quantify the effect of time on the perception of navigation. The perception of navigation is likely to improve as investigators gain more practice with the single-use rhinolaryngoscope.

### Environmental impact and cost

The incremental cost for each procedure is dependent on the clinical setting and the volume of procedures. We found that the new single-use rhinolaryngoscope was an effective and cost-minimising alternative in the acute surgical assessment unit, where the volume of procedures per rhinolaryngoscope was lower than in the out-patient clinic. The volume of procedures was previously shown to have a direct impact on the procedural cost for reusable endoscopes in a large teaching hospital in the UK.^22^ In accordance with McCahon and Whynes (2015), the high volume of procedures in the out-patient clinic pushed the capital and repair costs for reusable scopes below the cost of single-use rhinolaryngoscopes.^22^

• The single-use rhinolaryngoscope eliminates the serious potential risk of prion transmission in ENT endoscopy
• It provides comparable functionality to the reusable rhinolaryngoscope in terms of image quality, ergonomics, and advancing and navigation
• The single-use rhinolaryngoscope is a cost-minimising alternative in the acute surgical assessment unit, where volume of procedures per rhinolaryngoscope is lower than in an out-patient setting

Participants were also aware of the potential environmental impact of single-use rhinolaryngoscopes; comments included ‘have to empty/change bins and delay clinic as full up of used scopes – additional hospital costs re: change waste’ and ‘just used for rhinology/post-nasal space exam. Felt extravagant to use for that as not great view’. However, a previous study carried out a specific environmental comparison of single-use bronchoscopes in comparison with reusable bronchoscopes, and demonstrated that the single-use system had a similar environmental impact as reprocessing reusable bronchoscopes.^14^

### Study limitations

There were a number of limitations to our study that must be considered. First, despite our overall positive results, it is impossible to state conclusively that the single-use rhinolaryngoscope is better than the reusable rhinolaryngoscope without a comparative dataset. Consequently, there is a clear need for further research in this area. Second, the exact anatomical structures visualised during the procedures carried out by our investigators were not documented. Third, this study did not take the investigators’ learning curves into consideration; consequently the initial training session was not mandatory for all investigators. Fourth, the overall low level of compliance (29.5 per cent, 59 out of 200) demonstrated that the majority of procedures were not reported. This reduces internal and external validity, but also implies limitations in the range of procedures performed with the single-use rhinolaryngoscope. Fifth, a range of doctors participated in this study, with the vast majority being at the consultant level. Consequently, feedback was provided by doctors with a sufficient level of consolidated experience to enable appropriate comparison with the reusable rhinolaryngoscope. It is possible that reversal learning and the renewal effect, factors known to be involved in the adoption of new technology,^21^ might lead to a certain level of reluctance in doctors to adopt the single-use rhinolaryngoscope. Sixth, because of limited access to cost data, some of the costs used in our calculations were based on assumptions from cost analyses carried out in other UK hospitals. Therefore, the actual procedural costs within St George's University Hospital may differ from our base-case.

Finally, we must highlight the fact that the context (setting and indication) within which the data were obtained was not recorded in every feedback form. This is important as the desired functionality of the rhinolaryngoscope may change. For example, in an acute admission, it is very important to rapidly assess the overall state of the airway; in the out-patient department, priority is given to the assessment of finer details.

### Future recommendations

Future research should involve a comparative, prospective study, carried out in different clinical settings over a longer period of time, which includes the eyepiece, video and single-use rhinolaryngoscopes. Such work will be important in comparing the ability of these systems to clinically assess patients, and evaluate their overall impact at an organisational level. Research also needs to assess the impact of the portable image monitor on: the wider otolaryngology team, clinician learning, documentation, medical education and the patients’ perspective.

## Conclusion

The Ambu aScope 4 RhinoLaryngo Slim single-use rhinolaryngoscope provides a user experience that is clinically comparable to the reusable rhinolaryngoscope, at least with regard to all of the variables evaluated in this study. The perception of navigation had a significant influence on investigators’ opinions of whether this system can replace reusable rhinolaryngoscopes. In the acute surgical assessment unit, the Ambu aScope 4 RhinoLaryngo Slim single-use rhinolaryngoscope provides a cost-equivalent alternative to the reusable eyepiece rhinolaryngoscope, and a cost-minimising alternative to reusable video rhinolaryngoscopes.

## Supplementary material

For supplementary material accompanying this paper visit https://doi.org/10.1017/S0022215120001656. click here to view supplementary material

## References

[1] De Groen PC. History of the endoscope [scanning our past]. Proc IEEE 2017;105:1987–95

[2] Maqbool M, Maqbool S. Textbook of Ear, Nose & Throat Diseases, 11th edn. New Delhi: Jaypee Brothers, 2007

[3] Gov.UK. Health and Social Care Act 2008: code of practice on the prevention and control of infections. In: https://www.gov.uk/government/publications/the-health-and-social-care-act-2008-code-of-practice-on-the-prevention-and-control-of-infections-and-related-guidance [1 November 2019]

[4] Rutala WA, Weber DJ. New developments in reprocessing semicritical items. Am J Infect Control 2013;41:S60–6 23622752 10.1016/j.ajic.2012.09.028

[5] British Society of Gastroenterology. Guidance on Decontamination of Equipment for Gastrointestinal Endoscopy: 2017 Edition. In: https://www.bsg.org.uk/resource/guidance-on-decontamination-of-equipment-for-gastrointestinal-endoscopy-2017-edition.html [1 November 2019]

[6] Gov.UK. Decontamination of surgical instruments (HTM 01-01). In: https://www.gov.uk/government/publications/management-and-decontamination-of-surgical-instruments-used-in-acute-care [14 April 2020]

[7] Swift AC; ENT UK. Health Technical Memorandum HTM 01-06: Decontamination of Flexible Endoscopes and Rigid Endoscopes 2017. London: ENT UK, 2017

[8] ENTUK Guidelines for changes in ENT during COVID-19 Pandemic. In: https://www.entuk.org/entuk-guidelines-changes-ent-during-covid-19-pandemic [14 April 2020]

[9] Isle of Wight NHS Trust. Care, decontamination and maintenance of endoscopes and similar devices policy. In: https://www.iow.nhs.uk/Downloads/Policies/Care Decontamination and Maintenance of Endoscopes and Similar Devices Policy.pdf [4 December 2019]

[10] Selvadurai D. Using Nasoendoscopes Out Of Hours. St George's NHS Trust Local Policy. London: St George's University Hospitals NHS Foundation Trust, 2016

[11] Ofstead CL, Quick MR, Wetzler HP, Eiland JE, Heymann OL, Sonetti DA Effectiveness of reprocessing for flexible bronchoscopes and endobronchial ultrasound bronchoscopes. Chest 2018;154:1024–34 29859183 10.1016/j.chest.2018.04.045

[12] Ambu. ENT (Ear, Nose, Throat). In: https://www.ambu.com/products/ent [1 November 2019]

[13] Châteauvieux C, Farah L, Guérot E, Wermert D, Pineau J, Prognon P Single-use flexible bronchoscopes compared with reusable bronchoscopes: positive organizational impact but a costly solution. J Eval Clin Pract 2018;24:528–35 29573067 10.1111/jep.12904

[14] Sørensen BL, Grüttner H. Comparative study on environmental impacts of reusable and single-use bronchoscopes. Am J Environ Prot 2018;7:55–62

[15] Marshall DC, Dagaonkar RS, Yeow C, Peters AT, Tan SK, Tai DYH Experience with the use of single-use disposable bronchoscope in the ICU in a tertiary referral center of Singapore. J Bronchology Interv Pulmonol 2017;24:136–43 28323727 10.1097/LBR.0000000000000335

[16] Becker S, Hagemann J, O'Brian C, Weber V, Döge J, Helling K First experiences with a new flexible single-use rhino-laryngoscope with working channel - a preliminary study. Laryngorhinootologie 2019;98(S02):22–3

[17] Curtis LA, Burns A. Unit Costs of Health and Social Care 2017. Canterbury: Personal Social Services Research Unit, University of Kent, 2017;260

[18] Drummond MF, Schulpher MJ, Claxton K, Stoddart GL, Torrance GW. Method for the Economic Evaluation of Health Care Programmes. Oxford: Oxford University Press, 2015

[19] OECD. OECD Economic Outlook, Interim Report September 2019. Paris: OECD Publishing, 2019

[20] AMGROS Estimating unit costs. In: https://amgros.dk/media/2227/amgros-estimating-unit-costs.pdf [22 June 2020]

[21] Webster SJ, Bachstetter AD, Nelson PT, Schmitt FA, Van Eldik LJ. Using mice to model Alzheimer's dementia: an overview of the clinical disease and the preclinical behavioral changes in 10 mouse models. Front Genet 2014;5:88 24795750 10.3389/fgene.2014.00088 PMC4005958

[22] McCahon RA, Whynes DK. Cost comparison of re-usable and single-use fibrescopes in a large English teaching hospital. Anaesthesia 2015;70:699–706 25644476 10.1111/anae.13011

