# Supplementary material for: The single-use rhinolaryngoscope: an evaluation and cost comparison
Source: J Laryngol Otol. 2020 Sep 28;134(9):790–7. doi: 10.1017/S0022215120001656 (PMC7576370; doi:10.1017/S0022215120001656)
Supplement: Supplementary file 1 [file S0022215120001656sup001.doc]

<JLO 19347; supplementary material>

## **Table 1.** Capital equipment for out-patient clinic: eyepiece scenario

| Item | Capital cost 2018 (£) | Repair or service cost (per annum) (£) |
| --- | --- | --- |
| Eyepiece scope 1 | 4317 | 2000 |
| Eyepiece scope 2 | 4317 | 2000 |
| Eyepiece scope 3 | 4317 | 2000 |
| Eyepiece scope 4 | 4317 | 2000 |
| Eyepiece scope 5 | 4317 | 2000 |
| Eyepiece scope 6 | 4317 | 2000 |
| Eyepiece scope 7 | 4317 | 2000 |
| Eyepiece scope 8 | 4317 | 2000 |
| Eyepiece scope 9 | 4317 | 2000 |
| Eyepiece scope 10 | 4317 | 2000 |
| Eyepiece scope 11 | 4317 | 2000 |
| Eyepiece scope 12 | 4317 | 2000 |
| Eyepiece scope 13 | 4317 | 2000 |
| Eyepiece scope 14 | 4317 | 2000 |
| Eyepiece scope 15 | 4317 | 2000 |
| Eyepiece scope 16 | 4317 | 2000 |
| Eyepiece scope 17 | 4317 | 2000 |
| Eyepiece scope 18 | 4317 | 2000 |
| Eyepiece scope 19 | 4317 | 2000 |
| Eyepiece scope 20 | 4317 | 2000 |
| Eyepiece scope 21 | 4317 | 2000 |
| Eyepiece scope 22 | 4317 | 2000 |
| Eyepiece scope 23 | 4317 | 2000 |
| Eyepiece scope 24 | 4317 | 2000 |
| Eyepiece scope 25 | 4317 | 2000 |
| Eyepiece scope 28 | 4317 | 2000 |
| Eyepiece scope 27 | 4317 | 2000 |
| Eyepiece scope 28 | 4317 | 2000 |
| Light source 1 | 2212 | 2000 |
| Light source 2 | 2212 | 2000 |
| Light source 3 | 2212 | 2000 |
| Light source 4 | 2212 | 2000 |
| Light source 5 | 2212 | 2000 |
| Light source 6 | 2212 | 2000 |
| Light source 7 | 2212 | 2000 |
| Light source 8 | 2212 | 2000 |
| Light box 1 | 2212 |  |
| Light box 2 | 2212 |  |
| Stack machine | 16 806 | 2000 |

## **Table 2.** Capital equipment for out-patient clinic: video scenario

| Item | Capital cost 2018 (£) | Repair or service cost (per annum) (£) |
| --- | --- | --- |
| Videoscope 1 | 16 784 | 2000 |
| Videoscope 2 | 16 784 | 2000 |
| Videoscope 3 | 16 784 | 2000 |
| Videoscope 4 | 16 784 | 2000 |
| Videoscope 5 | 16 784 | 2000 |
| Videoscope 6 | 16 784 | 2000 |
| Videoscope 7 | 16 784 | 2000 |
| Videoscope 8 | 16 784 | 2000 |
| Videoscope 9 | 16 784 | 2000 |
| Videoscope 10 | 16 784 | 2000 |
| Videoscope 11 | 16 784 | 2000 |
| Videoscope 12 | 16 784 | 2000 |
| Videoscope 13 | 16 784 | 2000 |
| Videoscope 14 | 16 784 | 2000 |
| Videoscope 15 | 16 784 | 2000 |
| Videoscope 16 | 16 784 | 2000 |
| Videoscope 17 | 16 784 | 2000 |
| Videoscope 18 | 16 784 | 2000 |
| Videoscope 19 | 16 784 | 2000 |
| Videoscope 20 | 16 784 | 2000 |
| Videoscope 21 | 16 784 | 2000 |
| Videoscope 22 | 16 784 | 2000 |
| Videoscope 23 | 16 784 | 2000 |
| Videoscope 24 | 16 784 | 2000 |
| Videoscope 25 | 16 784 | 2000 |
| Videoscope 28 | 16 784 | 2000 |
| Videoscope 27 | 16 784 | 2000 |
| Videoscope 28 | 16 784 | 2000 |
| Stack machine 1 | 16 806 | 2000 |
| Stack machine 2 | 16 806 | 2000 |
| Stack machine 3 | 16 806 | 2000 |
| Stack machine 4 | 16 806 | 2000 |
| Stack machine 5 | 16 806 | 2000 |
| Stack machine 6 | 16 806 | 2000 |
| Stack machine 7 | 16 806 | 2000 |
| Stack machine 8 | 16 806 | 2000 |

## **Table 3.** Capital equipment for acute surgical assessment unit: eyepiece scenario

| Item | Capital cost 2018 (£) | Repair or service cost (per annum) (£) |
| --- | --- | --- |
| Eyepiece scope 1 | 4317 | 2000 |
| Eyepiece scope 2 | 4317 | 2000 |
| Eyepiece scope 3 | 4317 | 2000 |
| Eyepiece scope 4 | 4317 | 2000 |
| Eyepiece scope 5 | 4317 | 2000 |
| Eyepiece scope 6 | 4317 | 2000 |
| Eyepiece scope 7 | 4317 | 2000 |
| Eyepiece scope 8 | 4317 | 2000 |
| Light source 1 | 2212 | 2000 |
| Light source 2 | 2212 | 2000 |
| Light box | 2212 |  |
| Stack machine | 16 806 | 2000 |

## **Table 4.** Capital equipment for acute surgical assessment unit: video scenario

| Item | Capital cost 2018 (£) | Repair or service cost (per annum) (£) |
| --- | --- | --- |
| Videoscope 1 | 16 784 | 2000 |
| Videoscope 2 | 16 784 | 2000 |
| Videoscope 3 | 16 784 | 2000 |
| Videoscope 4 | 16 784 | 2000 |
| Videoscope 5 | 16 784 | 2000 |
| Videoscope 6 | 16 784 | 2000 |
| Videoscope 7 | 16 784 | 2000 |
| Videoscope 8 | 16 784 | 2000 |
| Stack machine 1 | 16 806 | 2000 |
| Stack machine 2 | 16 806 | 2000 |
| Stack machine 3 | 16 806 | 2000 |

## **Table 5.** Reprocessing costs for capital equipment

| Item | Capital cost 2018 (£) | Repair or service cost (per annum) (£) |
| --- | --- | --- |
| Manual leak tester 1 | 226 |  |
| Manual leak tester 2 | 226 |  |
| Manual leak tester 3 | 226 |  |
| Manual leak tester 4 | 226 |  |
| Manual leak tester 5 | 226 |  |
| Manual leak tester 6 | 226 |  |
| Soap dispenser 1 | 475* |  |
| Soap dispenser 2 | 475* |  |
| Soap dispenser 3 | 475* |  |
| Soap dispenser 4 | 475* |  |
| Soap dispenser 5 | 475* |  |
| Soap dispenser 6 | 475* |  |
| Endoscope re-processor 1 | 28 594 | 2495 |
| Endoscope re-processor 2 | 28 594 | 2495 |
| Endoscope re-processor 3 | 28 594 | 2495 |
| Endoscope re-processor 4 | 28 594 | 2495 |
| Endoscope re-processor 5 | 28 594 | 2495 |
| Endoscope re-processor 6 | 28 594 | 2495 |
| Quarterly validation contract |  | 15 258 |
| Drying cabinet out-patient | 20 995*^†^ | 2000*^†^ |
| Storage cabinet assessment unit | 1200 |  |
| Cleanascope 5 tray cart | 950 |  |
| Storascope with 10 trays | 2695 |  |
| Cleanascope 6 tray cart | 950 |  |
| Cleaning adaptors | 21 397 |  |
| Zink & counters | 6298*^†^ |  |
| Trollies & transport boxes | 1317 |  |
| Lids for transport boxes | 902 |  |
| Computer 1 | 600 |  |
| Computer 2 | 600 |  |
| Computer 3 | 600 |  |
| Tracking system | 94 477*^†^ | 30 000*^†^ |
| Reprocessing room £2500/m2 | 230 000*^†^ |  |
| Clean water plant | 27 293*^†^ |  |

*Assumptions based on UK hospital intelligence and costs available from online sources. ^†^Based on Mouritsen *et al*.^1^

**Table 6.** Single-use equipment and utilities

| Item | Cost per procedure (£) |
| --- | --- |
| Preparation of scope |  |
| – Apron | 0.84 |
| – Liners | 0.53 |
| – Handwash soap | 0.001* |
| Pre-cleaning |  |
| – Water | 0.01* |
| – Water disposal | 0.01* |
| – Tristal wipes | 1.3 |
| – Apron | 0.07 |
| – Gloves | 1.3 |
| – Liners | 0.53 |
| – PDI Sani cloth 70% | 0.13 |
| – PDI Sani cloth detergent wipes | 0.09 |
| – UniSept | 0.01* |
| – Paper towels | 0.01* |
| Dirty side manual cleaning |  |
| – Water | 0.15* |
| – Water disposal | 0.15* |
| – EndoZime AW Plus | 0.65 |
| – Apron | 0.07 |
| – Gloves | 1.30 |
| – Gauze swap | 0.16 |
| – Face shield | 1.89*^†^ |
| High-level disinfection |  |
| – Water | 0.01* |
| – Water disposal | 0.01* |
| – Electricity | 0.02*^‡^** |
| – Rapicide A | 1.00 |
| – Rapicide B | 1.00 |
| – Intercept Plus | 0.32 |
| – Printer paper | 0.12 |
| Clean side |  |
| – Apron | 0.07 |
| – Gloves | 0.06 |
| – Scope label (HESA) | 0.07 |
| – Liner | 0.53 |
| – Hook ups | 1.00 |
| Drying |  |
| – Electricity | 0.04 |
| Cleaning verification |  |
| – Protein test | 0.0002* |

*Assumptions based on UK hospital intelligence and costs available from online sources. Based on: ^†^Mouritsen *et al*. (2020);^1^ ^‡^Sørensen and Grüttner (2018);^2^ and **UKPower energy prices (2019).^3^ HESA = Health Edge Scope Application

**Table 7.** Personnel time and cost

| Parameter | Preparation of scope | Pre-cleaning | Transport 1 | Dirty side cleaning | Clean side | Transport 2 |
| --- | --- | --- | --- | --- | --- | --- |
| Time (mean ± SD; minutes) | 2.60 ± 0.11 | 6.97 ± 3.11 | 2.36 ± 0.97 | 6.17 ± 2.08 | 0.92 ± 0.63 | 2.28 ± 1.16 |
| Cost aSAU (mean ± SD; £) | 1.16 ± 0.05 | 3.10 ± 1.38 | 1.05 ± 0.43 | 2.74 ± 0.93 | 0.41 ± 0.28 | 1.01 ± 0.51 |
| Cost OPC (mean ± SD; £) | 2.14 | 2.14 | 2.14 | 2.74 ± 0.93 | 0.41 ± 0.28 | 2.14 |

SD = standard deviation; aSAU = acute surgical assessment unit; OPC = out-patient clinic

**Fig. 1.** Evaluation form for Ambu aScope 4 RhinoLaryngo Slim single-use rhinolaryngoscope.

<Refhead>

References

<Reftext>

1 Mouritsen JM, Ehlers L, Kovaleva J, Ahmad I, El-Boghdadly K. A systematic review and cost effectiveness analysis of reusable vs. single-use flexible bronchoscopes. *Anaesthesia* 2020;**75**:529–40

2 Sørensen BL, Grüttner H. Comparative study on environmental impacts of reusable and single-use bronchoscopes. *Am J Environ Prot* 2018;**7**:55–62

3 UKPower. Compare Energy Prices Per kWh. In: https://www.ukpower.co.uk/home_energy/tariffs-per-unit-kwh [10 November 2019]
